# Formulation and Evaluation of Indomethacin Nanosuspensions Stabilized by Poly(2-oxazine) and Poly(2-oxazoline)-Based Polymers for Solubility Enhancement

**DOI:** 10.1007/s11095-026-04109-0

**Published:** 2026-05-15

**Authors:** Erika Espo, Dipti Potdar, Kiana Baas, Larissa Keßler, Mengshi Yang, Kārlis Bērziņš, Marianna Kemell, Topias Kiiskinen, Anni Heinonen, Josef Kehrein, Ben J. Boyd, Anssi-Pekka Karttunen, Leena Peltonen, Robert Luxenhofer, Alex Bunker, Tapani Viitala

**Affiliations:** 1 Division of Pharmaceutical Chemistry and Technology, Faculty of Pharmacy, University of Helsinki, FI-00014 Helsinki, Finland; 2 Division of Pharmaceutical Biosciences, Faculty of Pharmacy, University of Helsinki, FI-00014 Helsinki, Finland; 3 Laboratory of Pharmaceutical Technology, Department of Pharmaceutics, Ghent University, Ottergemsesteenweg 460, 9000 Ghent, Belgium; 4 Soft Matter Chemistry, Department of Chemistry, and Helsinki Institute of Sustainability Science, Faculty of Science, University of Helsinki, FI-00014 Helsinki, Finland; 5 Lehrstuhl Für Chemische Technologie Der Materialsynthese, Department of Chemistry and Pharmacy, Julius-Maximilians-University Würzburg, Röntgenring 11, 97070 Würzburg, Germany; 6 Department of Pharmacy, Faculty of Health and Medical Sciences, University of Copenhagen, 2100 Copenhagen, Denmark; 7 Department of Chemistry, Faculty of Science, University of Helsinki, FI-00014 Helsinki, Finland; 8 Institute of Pharmacy and Food Chemistry, University of Würzburg, Am Hubland, 97074 Würzburg, Germany; 9 Drug Delivery Disposition and Dynamics, Monash Institute of Pharmaceutical Sciences, Monash University, Victoria, 3052 Australia; 10 Pharmaceutical Sciences Laboratory, Faculty of Science and Engineering, Åbo Akademi University, FI-20500 Turku, Finland

**Keywords:** dissolution profile and dissolution rate enhancement, molecular dynamics simulations, nanosuspension, nanosuspension stability, poly(2-oxazoline) and poly(2-oxazine) polymers

## Abstract

**Objective:**

The challenges associated with poorly water-soluble drugs have been recognized for several decades. Poor aqueous solubility directly impairs the bioavailability of a drug, but this can be significantly improved using a nanosuspension (NS) formulation strategy. In general, there is high variability in stabilizers that function with different drugs, and it is often difficult to predict which stabilizer will yield a stable NS formulation.

**Methods:**

In this study, poly(2-oxazine)- and poly(2-oxazoline)-based triblock copolymers (P1 and P2) were evaluated and benchmarked against commonly used stabilizers for the preparation of NS formulations, specifically hydroxypropyl methylcellulose and Pluronic F68. This study initiates the validation of P1 and P2 polymers for a new application, as these materials have not been previously studied as stabilizers in oral NS formulations. Indomethacin was selected as a poorly water-soluble model drug for NS preparation using a wet-ball milling technique. The stability of the resulting NSs was monitored over 28 days and their dissolution profiles were assessed under non-sink conditions.

**Results:**

P1 and P2 demonstrated better particle size reduction, and stability properties compared to the traditional stabilizers. In particular, P1 presented a marked improvement in the dissolution profiles of indomethacin, significantly outperforming the other NS formulations and reference samples. Molecular dynamics simulations further revealed distinct differences in the interactions of P1 and P2 with the indomethacin crystal surface, supporting the experimental findings.

**Conclusions:**

Overall, our study highlights the potential of P1 as a promising stabilizer for nanosuspensions, providing mechanistic insights into polymer-drug compatibility, improved dissolution performance, and formulation stability.

**Graphical Abstract:**

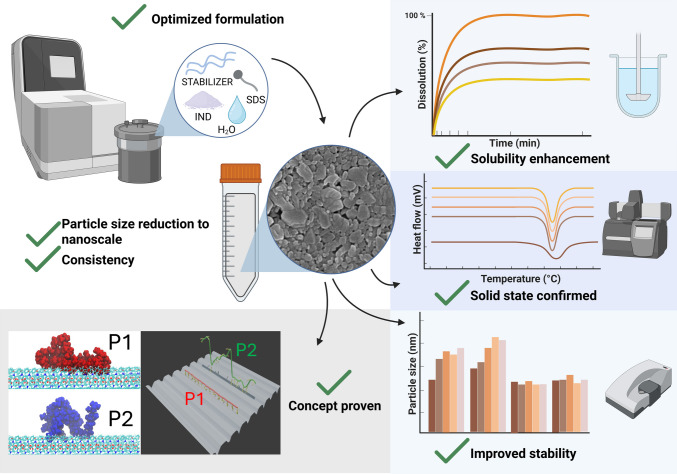

**Supplementary Information:**

The online version contains supplementary material available at 10.1007/s11095-026-04109-0.

## Introduction

Poor water solubility of drug compounds has become a major obstacle hampering modern drug development. It has been recognized for several decades that a large proportion of new active pharmaceutical ingredients (APIs) entering the market are classified as poorly water soluble [1, 2]. Poor water solubility is often linked to reduced bioavailability of the drug, and this challenge has consequently driven scientists and the pharmaceutical industry to seek new ways of overcoming this issue [3, 4].

The techniques and strategies for preparing drug nanosuspensions (NSs) are well known. Compared to many other nanotechnology approaches under research, this is a relatively simple, well-established formulation strategy. It improves the dissolution profiles and solubility of drugs, even in situations where the solubility is poor, or the drug is considered insoluble in water [2, 5, 6]. Nevertheless, this nanosizing strategy still has its own challenges such as suboptimal colloidal stability of suspensions of drug crystals, complexity of finding the optimal stabilizer, and limitations related to routes of administration [7, 8]. The risk of embolism during intravenous administration of NSs cannot be ruled out, but the other above-mentioned challenges could to some extent be addressed with NSs, especially in the case of oral administration.

The solubility enhancement achieved using NSs, *i.e.,* dispersed drug nanocrystals, is based on increasing the overall surface area of the drug crystals, and hence significantly amplifying interactions between the drug crystals and the solvent causing an improved dissolution rate [6]. This phenomenon is supported by the Kelvin-Thomson equation which states that solubility improves significantly when the particle size is decreased to the nanoscale (< 1 µm). The increased surface area challenges classical thermodynamic assumptions and, thus, alters the characteristics of the drug particle. Accordingly, NS formulations generate a higher supersaturation level of the drug compound when compared to the bulk material if the nanosizing of the drug particle is maintained. Importantly, when decreasing the particle size to only a few hundreds of nanometers requires that the size distribution of the drug particles must be somehow stabilized to avoid Ostwald ripening, aggregation, and sedimentation of the drug particles [9]. Stabilizers that are often used for NS formulations are for example polysorbates (Tween® series), block copolymers (*e.g*., Pluronics®) and hydroxypropyl methylcellulose (HPMC) [10, 11]. For this study, indomethacin (IND) was chosen as a model API, since its water-solubility has been reported to be very poor or practically insoluble (Biopharmaceutical Classification System (BCS) class II), and it has been shown that a NS formulation strategy can be used to enhance the dissolution characteristics of the drug [12, 13]. Firstly, the colloidal stability of IND NS formulations was screened using different polymer-based stabilizer systems, starting with two traditionally used polymer stabilizers, *i.e.*, Pluronic® F68 (hereinafter F68) and HPMC to optimize the preparation method and formulation strategy. Then, the optimized preparation method and formulation strategy were implemented with poly(2-oxazine) (POzi) and poly(2-oxazoline) (POx)-based amphiphilic triblock copolymers, herein referred to as P1 and P2, as novel stabilizers for NS formulations. Our aim was to determine the potential of POzi and POx-based polymers as stabilizers for orally administered NSs of poorly soluble drugs.

**Fig. 1:**
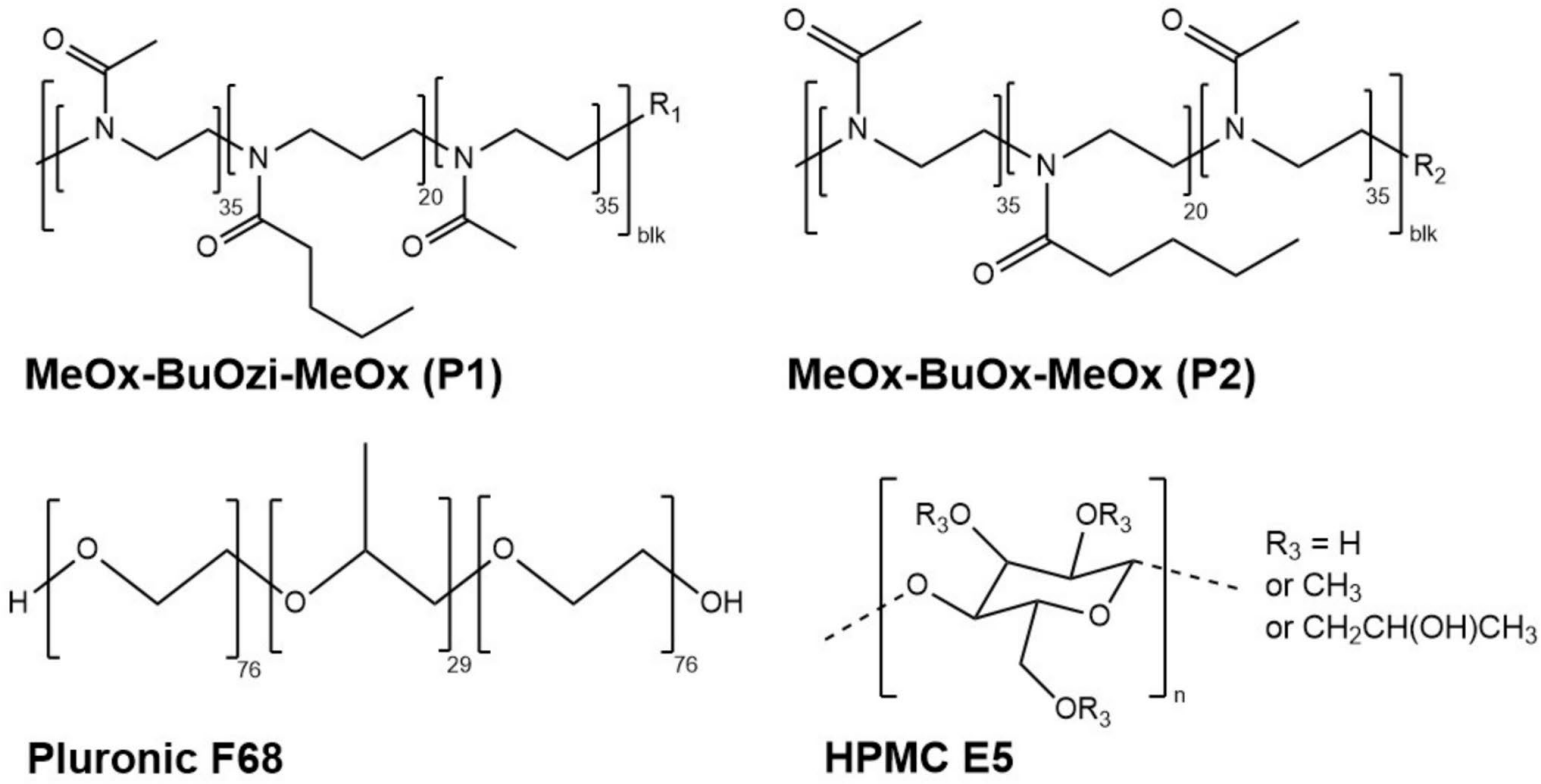
Molecular structures of P1 and P2, and traditional stabilizers F68 and HPMC used as comparators. R_1_ abbreviates ethyl isonipecotate and R_2_ piperidine.

The structures of P1 and P2 (Fig. 1) were inspired by the general Pluronic® structure, insofar as they are ABA triblock copolymers with highly hydrophilic A blocks and a hydrophobic B block. The structures of P1 and P2 differ in the nature of the middle B block. In P1, the middle block is synthesized from a 2-butyl-2-oxazine monomer, while P2 comprises a 2-butyl-2-oxazoline based central block. The additional methylene group in the backbone of P1 increases the backbone flexibility, thus reducing the glass transition temperature. These types of polymers have previously shown promising potential in improving the solubility of a wide variety of poorly water-soluble drugs in micellar drug delivery system applications, as well as shown promising results as polymers for preparing amorphous solid dispersion formulations with IND [14–18]. More recently, the effect of drug loading into polymer micelles with these structures on the interactions with simulated intestinal fluids was studied [19, 20]. It should be noted that POx or POzi are not considered readily biodegradable. Potential hydrolysis in the digestive tract would likely be too slow to be considerable [21], however, oxidative degradation by reactive oxygen species has been described but considered unlikely to be relevant after oral administration [22]. Additionally, POx-based amphiphilic triblock copolymers such as P2 have shown excellent cyto- and biocompatibility [23–26]. For POzi-based polymers like P1, less safety data is available, but all available data shows excellent cytocompatibility [27–29]. Important to note, Ramsey *et al.* [30] have already initiated the study of using extremely low concentrations of P2 as a stabilizer for sub-micron-sized formulations of remdesivir for aerosol treatment of COVID-19. However, the aim of the present study was to assess the use of P1 and P2 polymers as stabilizers for smaller particles of only a few hundred nanometers and elucidating their ability for colloidal stabilization compared to other stabilizer systems. In addition, molecular dynamics (MD) simulations were conducted to investigate the interaction mechanisms between the drug particles and POzi and POx-based polymers on the atomic level, analyzing the spatial arrangement of POzi and POx-based polymers on the drug crystal surface.

Moreover, a formulation challenge for the development of NS formulations is the limitations of the acceptable levels of excipients, such as surfactants in drug preparations [31]. Charged surfactants like sodium dodecyl sulphate (SDS), when used in combination with polymer stabilizers, are often employed to improve colloidal stability of NSs of poorly soluble drugs to enhance their dissolution rate [32, 33]. These surfactants are commonly used alongside non-ionic polymers, as they can occupy the spaces between polymer chains and enhance electrostatic repulsion between particles [34]. This dual stabilization mechanism often improves the colloidal stability of NS formulations. In such systems, the surfactants may contribute an additional electrostatic repulsion component to the steric stabilization provided by the non-ionic polymers. However, this contribution is not necessarily required for all polymer types, as the effectiveness of stabilization can depend on the specific characteristics of the polymer used. Therefore, we also investigated here whether SDS could improve the stability and dissolution properties of the polymer-stabilized NS formulations.

## Material and Methods

### Materials

Indomethacin (IND) was purchased from Tokyo Chemical Industry Co., Ltd. (Tokyo, Japan). Poloxamer 188 (Pluronic® F68) was purchased from BASF Co. (Ludwigshafen, Germany). Hydroxypropyl methylcellulose (HPMC) E5 was purchased from Dow Chemical Company (Midland, Michigan, USA). Sodium dodecyl sulfate (SDS) and potassium biphthalate were purchased from Sigma-Aldrich® (St. Louis, USA). Sodium hydroxide was purchased from VWR® Chemicals, LLC (Zwijndrecht, Belgium). Ion exchanged water (18.2 MΩ·cm, ≤ 2 ppb TOC) (Milli-Q IQ 7005 Ultrapure, Merck, Darmstadt, Germany) was used in all experiments that included water.

### Polymer Structures

The synthesis of the ABA triblock copolymers P1 and P2 has previously been described, *inter alia* by Haider and Luxenhofer [14]. The same batch of polymers P1 and P2 as described in a previous study by Keßler and Luxenhofer [35] was used in this work. P1 is composed of 2-methyl-2-oxazoline (MeOx) A blocks and a 2—*n*-butyl-2-oxazine (BuOzi) B block, and 2—*n*-butyl-2-oxazoline (BuOx) is used in the structure of P2 instead of BuOzi (Fig. 1). More specifically, P1/P2 features A blocks of slightly greater than 3 kg/mol and a hydrophobic B block between 2 and 3 kg/mol. The ratio in the structures is very similar to the reference stabilizer F68.

### Preparation of Nanosuspensions

Various amounts of polymer stabilizer and surfactant were dispersed in 5 mL of water. The amount of polymer stabilizer was varied between 25 and 100 mg (Table S1). Similarly, the SDS content, when included, was varied between 2.5 and 10 mg. The formulation composition codes indicate drug + [stabilizer](mass in milligrams) + SDS(mass in milligrams). IND (1.25 g) was mixed with the stabilizer solution. The mixture was transferred on top of 30 g zirconium oxide milling balls (diameter 0.5 mm) in the milling vessel. The particle size reduction to nanoscale was performed by wet ball milling (Pulverisette 7 Premium, Fritsch Co., Idar-Oberstein, Germany). The IND-stabilizer mixtures were milled (1100 rpm) for 30 min in total. Milling was implemented in a 3-min milling cycle with 15-min cooling breaks. Cooling of milling vessels was carried out by placing the intact vessel into an ice bath. The prepared NSs were visually inspected and collected for further studies. After preparation, samples were stored in a refrigerator at + 5°C.

### Particle Size and Zeta Potential

Particle size, polydispersity index (PdI) and zeta (ζ)-potential were measured using dynamic light scattering (DLS) and electrophoretic mobility, respectively (Zetasizer ZS Nano, Malvern Instruments Ltd., Worcestershire, UK). All measurements in this study were conducted at 25°C and the viscosity of the dispersant was set to 0.8872 cP. Target particle size and PdI on the preparation day were set to be less than 350 nm and below 0.3, respectively. The particle size limit was set to ensure the nanoscale dimension of the particles and the PdI value was set to ensure that the NSs were homogenous supporting the stability of the NSs and to exclude any possible effects in the dissolution tests caused by significantly different particle sizes. A limit for ζ-potential was not set as it is highly dependent on the used stabilizer.

Prior to the DLS measurements, the samples were diluted with drug-saturated water solution to prevent dissolution impacting on the particle size distribution. Briefly, the NS sample was mixed using a vortex mixer and then diluted with the saturated drug solution in a 1:19 v/v ratio. The diluted solution was gently mixed and then diluted again in a ratio of 1:30 v/v. The final diluted solution was placed in the cuvette and the particle size distribution was measured by using DLS.

The saturated drug solution was prepared in advance by dissolving 0.1 g of stabilizer in 100 mL of water and adding excessive amount of IND. Before use, the saturated solution was filtered with a 25 mm cellulose acetate membrane syringe filter with a 0.45 µm pore size (VWR, Pennsylvania, USA).

### Solid State Analysis

Solid-state analysis was performed using differential scanning calorimetry (DSC) and low-frequency Raman (LF-Raman) spectroscopy. DSC measurements were performed using DSC 823e (Mettler Toledo Inc., Greifensee, Switzerland). The NS samples (20 µL) were dried at 40°C in a perforated aluminum pan and sealed when the sample was confirmed to be completely dry. The reference samples were dry powders of the compounds used in the NS formulations which were directly weighed into the aluminum pan and sealed for DSC measurements. The temperature range was set to 25–180°C, the heating rate was 20°C/min and nitrogen gas was purged at a flow of 50 mL/min. NS samples were dried on the preparation day and on day 28 after the preparation.

LF-Raman experiments were performed using a Raman system (Ondax Inc., Monrovia, California, USA) with a 785 nm laser source (Ondax Inc., Monrovia, California, USA). NSs were studied within one week after preparation. NS samples were partially dried on top of an aluminum pan at room temperature immediately before the Raman spectrum was collected. In the same manner, dry reference samples were placed onto the aluminum pan. During data collection, the sample and the LF-Raman setup was covered with a black plastic cover to minimize the interference from ambient light emissions. Exposure time of 0.5 s was used for the acquisition of each spectrum as an average from 120 scans. Backscattered light (180° scale) was collected using a non-confocal optical assembly and filtered through a volume Bragg grating (Ondax Inc., Monrovia, California, USA). The backscattered light was focused into the spectrograph (Ondax Inc., Monrovia, California, USA) via a fiber-optic cable and dispersed onto a CCD detector (Andor iVac 316, Oxford Instruments, Abingdon, UK).

### Scanning Electron Microscopy

Scanning electron microscopy (SEM) images were acquired with a Hitachi S-4800 FESEM (field emission scanning electron microscope) (Hitachi High-Tech, Tokyo, Japan). Before imaging, the NSs were dried on top of a silicon wafer over-night at room temperature. All samples were coated with a 5 nm layer of Au–Pd alloy using a Cressington 208HR sputter coater (Cressington Scientific Instruments, Watford, UK).

### Dissolution Test

The evaluation of the dissolution profiles was performed under non-sink conditions to distinguish the differences between the NS formulations [36]. The dissolution tests were performed using the paddle method (Erweka DT-06, Heusentamm, Germany) and phthalate buffer (pH 5.0) as a dissolution medium. The volume of dissolution medium used was 600 mL. The amount of NS sample added to the dissolution vessel was calculated prior to the dissolution test from the saturated solubility of IND (21.23 µg/mL) at pH 5.0 (37°C), corresponding to the IND amount of 12.7 mg [36]. The paddle speed was set to 50 rpm. The NS sample was placed in the dissolution medium (37 ± 0.5°C) and sampled at pre-determined time points of 0.5, 1, 2, 4, 6, 15 and 30 min. The volume of the dissolution sample withdrawn from the dissolution vessel at each time point was 5 mL, and the removed volume was replaced with fresh pre-warmed buffer medium.

The drug concentration in the dissolution samples was measured using a UV − Vis spectrophotometer (UV-1600PC UV − Vis spectrophotometer, VWR, Pennsylvania, USA) at 318 nm. Prior to the measurements, each sample was filtered with a 25 mm cellulose acetate membrane sterile syringe filter with a 0.22 µm pore size (VWR, Pennsylvania, USA). Sample concentration was converted from the absorbance by using a standard curve (R^2^ = 0.9994).

### Molecular Dynamics Simulations

#### Polymer Setup

For molecular dynamics (MD) simulations, two truncated ABA-type triblock copolymers, P1 and P2, were constructed, each comprised of 18 repeating units of MeOx as the hydrophilic blocks (Block A) on both sides and 10 repeating units of BuOzi (for P1) or BuOx (for P2) as the central hydrophobic block (Block B). The end groups of the polymers were defined to match those used in the experimental work (see Fig. 1). Reduced polymer chain lengths were selected to ensure sufficient conformational sampling and to provide statistically robust data for both qualitative and quantitative analysis. Structures were prepared within Schrödinger Maestro 2025—2, geometry optimized within Avogadro 1.2.0 [37, 38], and submitted to the PolyParGen webserver for parameterization based on the Optimized Potentials for Liquid Simulations (OPLS-AA) force field [39–41]. The resulting polymers were energy minimized in vacuum using the steepest descent method, followed by a 10 ps NVT simulation at 300 K using the V-rescale thermostat (τ = 0.1 ps) with GROMACS 2024.3 [42, 43].

#### Indomethacin Setup

The crystal structure of γ-IND was obtained from the Cambridge Structural Database (CCDC refcode: INDMET03, space group P-1) [44]. Symmetry mates within a radius of 100 Å were initially generated using PyMOL 2.1 [45]. Subsequently, a crystal slab with dimensions of 15× 15 × 3 nm^3^ was built using the TopoTools package in VMD, resulting in a total of 1350 IND molecules [46]. Topology and parameters for individual IND molecules were generated using the LigParGen server with the OPLS-AA force field [40]. The crystal was solvated in TIP3P water [47], energy minimization was achieved using the steepest descent algorithm. The system was then equilibrated under NVT conditions for 300 ps at 300 K using the velocity rescale thermostat (τ = 0.1 ps) [43]. This was followed by NPT equilibration for 1000 ps with semi-isotropic pressure coupling allowing pressure scaling along the z-axis and using the V-rescale thermostat (τ = 0.1 ps) and C-rescale barostat (τ = 5.0 ps) with a compressibility of 4.5 × 10^−5^ bar^−1^ [43, 48].

#### Polymer-indomethacin Simulations

The equilibrated structures of polymers and the drug crystal were used to construct combined systems for further simulations to study drug-crystal interactions. Two systems were prepared: one with polymer P1 and one with polymer P2. Three independent replicas were generated for each system. Polymers were initially positioned approximately ~ 3 nm above the crystal surface with random orientation using the gmx insert-molecules command. The simulation box measured 20 nm along the z-axis, with the 3 nm thick IND crystal slab positioned in the x—y plane through the box center. All combined systems were solvated with TIP3P water; energy was minimized using a soft-core potential to remove potential clashes followed by conventional steepest descent minimization [49].

Systems were then relaxed under NVT conditions at 300 K for 10 ps (τ = 0.1 ps), followed by NPT equilibration under semi-isotropic pressure coupling using the V-rescale thermostat (τ = 0.1 ps) and C-rescale barostat (τ = 5.0 ps) for 5000 ps. Position restraints with a force constant of 1000 kJ mol^−1^ nm^−2^ were applied to IND crystal molecules throughout equilibration and production runs to model a solid crystal surface. Simulations were conducted using a 2 fs time step, and long-range electrostatic interactions were handled using the Particle Mesh Ewald (PME) method [50, 51]. The LINCS algorithm was applied to constrain the H-bonds [51]. Production MD simulations were performed with the same NPT parameters for 400 ns, saving trajectories every 100 ps for all three replicas of each polymer. Periodic boundary conditions were applied in all three directions for all simulations.

#### Trajectory Analysis

Trajectory visualization was performed using VMD 1.9.4 [52], and key structural and dynamic parameters were calculated using built-in GROMACS analysis tools and the MDAnalysis 2.9.0 package [53]. The analyses included the radius of gyration (R_g_), root-mean-square deviation (RMSD) and solvent-accessible surface area (SASA) of the polymers. SASA was computed using a probe radius of 0.14 nm. Contact analyses were performed to study polymer-crystal interactions. For this, MDAnalysis was used to identify hydrophobic–hydrophobic interactions using a 0.4 nm cutoff, primarily involving carbon atoms, and polar–polar interactions, focusing on oxygen and nitrogen atoms in both polymer blocks A and B for both polymers. Mass density profiles were generated for Block A and Block B of polymers P1 and P2 along the z-axis, perpendicular to the crystal surface, with the surface chlorine atoms of IND defined as the z = 0 reference plane. The simulation box was divided into 100 bins to enable accurate profiling. Box plots for contact analysis and mass density profile curves represent averaged data from production simulations of three replicas for the last 100 ns (*i.e*., from 300 to 400 ns) of each polymer after the initial relaxation phase (see Supplementary material Figure S4). The evolution of the polymer–surface center-of-mass (COM) separation (ΔZ) (Figure S4) shows an initial approach phase followed by a plateau region, indicating that the systems reach a stationary interfacial regime within the simulation timescale.

### Stability Studies

The physical stability evaluation was performed on day 1, 7, 14 and 28 after NS preparation. Particle size, PdI-value and ζ-potential were measured up to 28 days after NS preparation to evaluate the capability of the formulations to maintain their nanosized particle size. Measurements were performed in the same manner as described in the Particle Size and Zeta Potential section above.

### Statistical Analysis

The graphs were prepared using OriginPro® (OriginLab Corporation, Northampton, USA). The measured values are presented as mean values ± standard deviation of at least three replicates, except for DSC and LF-Raman measurements, which were performed as single measurements. DLS measurements were performed as technical replicates, while the dissolution tests were conducted using independent replicates. NS preparation is generally considered to be highly reproducible, and therefore the use of technical replicates for DLS measurements was deemed appropriate [2]. Nevertheless, it should be noted that this approach somewhat limits the scope of statistical inference. Statistical analysis was performed using the student t-test and statistical relevance was established at a *p*-value threshold of < 0.05.

## Results

### Pre-assessment of Formulations

Immediately after preparing the NS samples, they were visually inspected and measured by DLS to determine the particle size and PdI. Prior to subsequent experiments, these factors were evaluated to select suitable samples and to determine an appropriate threshold for the required amounts of reference stabilizers (F68 and HPMC), which were then considered for the P1 and P2 polymer formulations.

IND NS formulations were first screened with the traditional polymers F68 and HPMC and in combination with SDS to evaluate the effect of added SDS (Figure S1A-B). These two reference stabilizers at 100 mg (always per 1.25 g of IND) enabled production of NSs meeting the target characteristics (particle size < 350 nm, PdI < 0.3) even in the absence of added SDS. IND NSs with 100 mg of F68 and HPMC without SDS were found to have slightly larger mean particle sizes compared to the SDS containing formulations. On the day of preparation, IND + F68(100) and IND + HPMC(100) had mean particle sizes of 309 ± 0 nm and 331 ± 5 nm, respectively. In these formulations, addition of a small amount of SDS, *i.e.*, 2.5 mg, significantly reduced the particle size (p < 0.05) as can be seen for IND + F68(100) + SDS(2.5) and IND + HPMC(100) + SDS(2.5) which presented, on the day of preparation, mean particle sizes of 236 ± 2 nm and 231 ± 1 nm, respectively.

Reduction of the amount of reference stabilizer to 50 mg resulted in increased particle size and in some cases failed to meet the target criteria. NS formulations with 50 mg of F68 and HPMC showed strong variation in particle size distribution depending on the SDS concentration. F68 and HPMC formulations with 5 and 10 mg SDS were successfully prepared and fulfilled the target particle size and PdI. No difference in the formulations was found by increasing the amount of SDS from 5 to 10 mg. Formulations with the lowest SDS concentration, *i.e.*, IND + F68(50) + SDS(2.5) and IND + HPMC(50) + SDS(2.5), were rejected from further studies because they exhibited a large particle size and appeared non-homogenous even upon visual inspection of the suspensions. Also, IND NSs with 50 mg of F68 and HPMC without SDS confirmed that lowering the amount of stabilizer would not present a desired outcome without the addition of SDS into the formulations. NS formulations consisting of 25 mg of F68 and HPMC with SDS were also rejected from further studies as the particle sizes were too large, hence they did not comply with the target values. Notably, IND + HPMC(25) + SDS(10) achieved the target size. However, it formed a sample with a highly viscous, paste-like consistency, which significantly impaired the handling of the sample and was also excluded from further studies.

After the screening results for the reference stabilizers, same concentrations of P1 and P2 polymers (100 mg, 50 mg and 25 mg) were used and combined with the highest concentration of added SDS (10 mg) to evaluate possible effects of the added surfactant (Figure S1C-D) [54]. Plain P1 and P2 formulations complied with the target particle size and PdI. A noteworthy observation was that SDS was ineffective in the formulations with the highest studied amount of POzi and POx-based polymers regarding the particle size or homogeneity compared to the case with the reference stabilizers. In case of P2, the addition of SDS resulted in the sample failing to comply with the target values in the formulations with 50 mg of polymer. In contrast, for the P1 formulation, the addition of SDS (10 mg) ensured a small particle size and homogeneity of the samples even with the lowest amount of POzi-based polymer (25 mg). Nevertheless, IND + P1(25) + SDS(10) did not comply with the target values due to an excessively high PdI-value. Since formulations with 50 mg of stabilizer, even when combined with the highest studied SDS concentration, did not meet the preliminary requirements, further studies were proceeded with NS formulations with the highest amount of stabilizer (100 mg).

### Solid-state Analysis

**Fig. 2:**
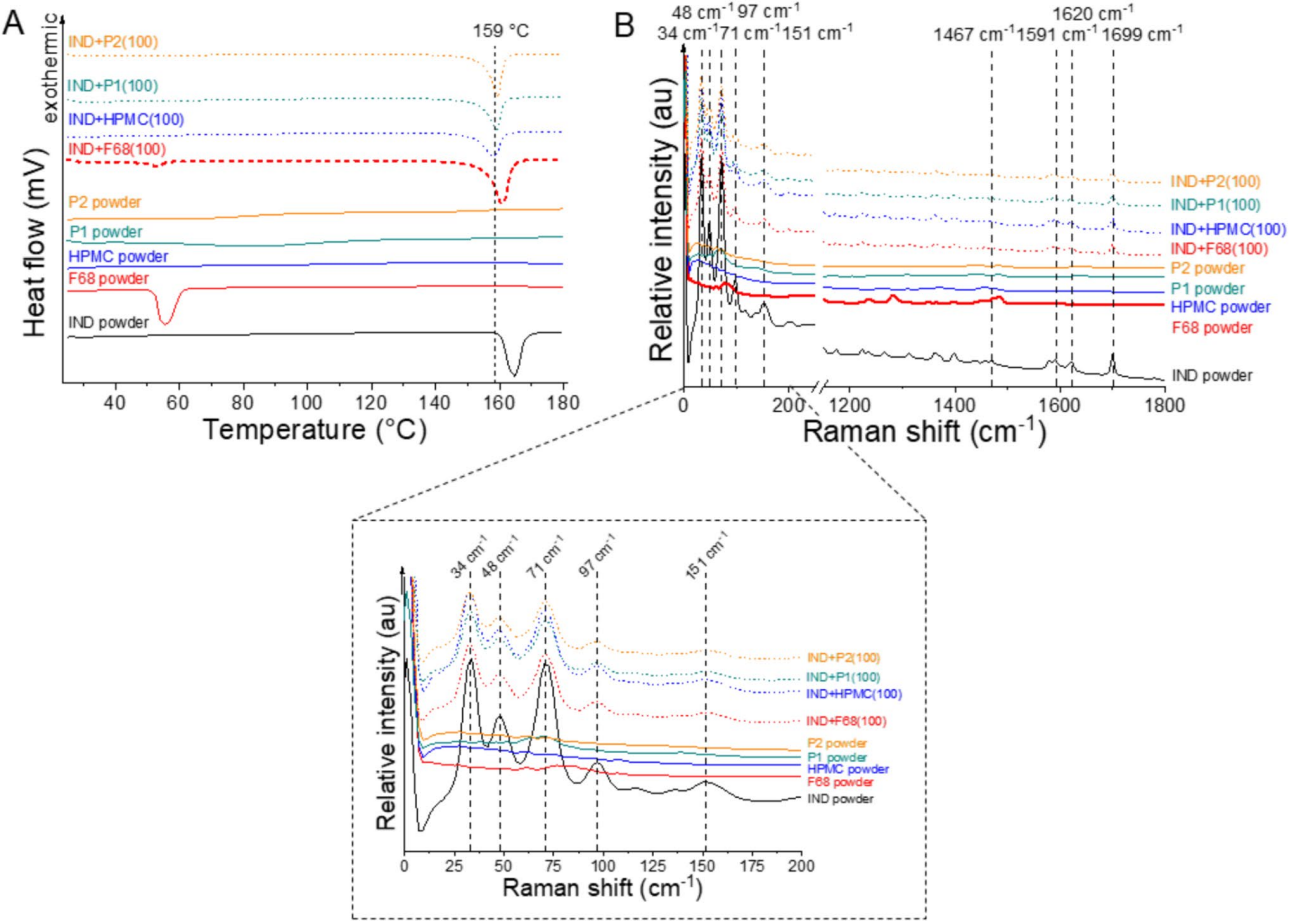
Impact of formulation and stabilizer on thermal behavior and solid-state form of IND NSs. (**A**) DSC thermograms for NS formulations and reference samples (*n* = 1). Dashed vertical line indicates onset of melting temperatures of powder IND. (**B**) LF-Raman spectra of NSs and reference samples. Dashed vertical lines highlight the peaks that are typical for the γ-form of IND.

The onset of the melting temperature of pure IND was determined to be 159°C, which provided an initial indication of the crystalline gamma (γ) solid-state form of IND (Fig. 2A). However, in physical mixtures of powders of the stabilizers and IND, the onset of the melting temperature decreased to 152–153°C. This was an expected finding because the addition of polymers increases the viscosity of the mixture, thus affecting the overall molecular mobility [55, 56]. Furthermore, once the excipients have melted, IND can dissolve into them, which may also contribute to the observed shift in the melting point [57]. The melting point depression may be observed due to apparent impurity of the sample when compared to the pure bulk material. The onset of the melting temperature was in the range of 145°C to 152°C for all NS formulations. In the case of NSs, the reason may also be that a smaller particle size sample requires less thermal energy for phase transition. The onset of the melting temperature did not significantly change (p > 0.05) for any of the NS formulations during storage over 28 days, which indicates that no structural changes or significant degradation of IND occurred during storage (Figure S2). Based on the DSC results, IND was in a crystalline form across all NS formulations. No glass transition events were observed, even upon closer inspection, further supporting that IND persisted in the crystalline state.

In addition to the DSC measurements, LF-Raman measurements supported the crystalline state of IND being the same as the reference crystalline starting material (Fig. 2B). Peaks in the range between 1500 cm^−1^ and 1750 cm^−1^ indicate a stretching of the carbonyl group and the benzyl group of IND present in the crystalline reference and the prepared NS samples [58, 59]. The LF-Raman range from 0 cm^−1^ to 250 cm^−1^ indicates the lattice vibration of samples [60]. Especially, the so-called ‘phonon modes’ in the LF-Raman wavenumber range are indicative of the presence of a long-range order. Based on the observations, the Raman peaks found in the spectra are characteristic of the γ-form of IND [61]. Additionally, phonon peak characteristics were compared among the samples, as phonon confinement can occur when the dimensions of a crystal approach the phonon wavelength. Although the crystallite sizes in the NS formulations (discussed below) were relatively large for strong confinement effects, partial manifestations such as peak broadening, changes in relative peak intensities, and peak red-shifting can still be observed. However, in all cases, the phonon peak profiles remained consistent across the NS samples, indicating no significant confinement-related spectral changes.

### Scanning Electron Microscopy

**Fig. 3:**
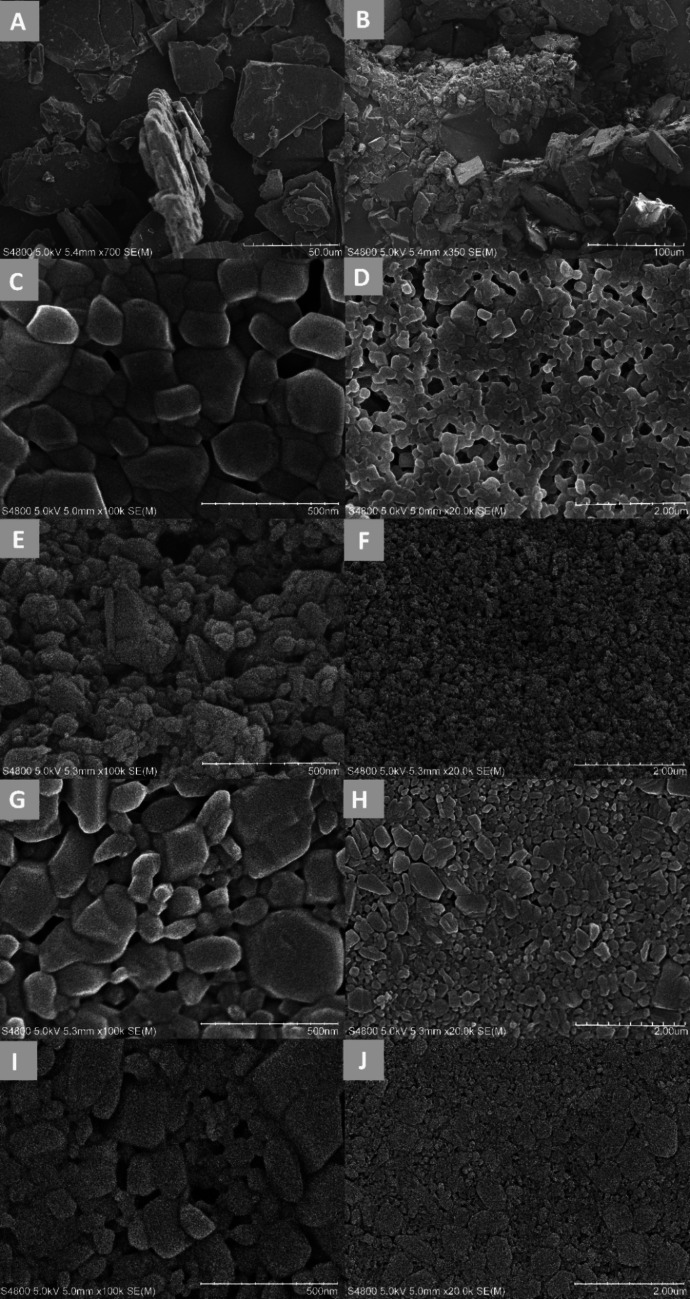
SEM images of (**A**) and (**B**) IND powder (scale bars 50.0 µm and 100 µm, respectively), (**C**) and (**D**) Dried IND + F68(100), (**E**) and (**F**) IND + HPMC(100), (**G**) and (**H**) IND + P1(100), and (**I**) and (**J**) IND + P2(100). Scale bars for NSs are 500 nm (left images) and 2.00 µm (right images).

Pristine IND powder and dried NS samples were investigated using SEM (Fig. 3). The morphology of IND powder was clearly different, and as expected, the particle size was significantly larger for IND powder compared to NSs. However, the IND + HPMC (Fig. 3E-F) presented different morphologies compared to the other NSs. For IND + HPMC, the IND drug crystals appear to be unevenly covered with HPMC. HPMC is seemingly aggregated on top of the drug crystals, whereas the other stabilizers appear to have an even coating as judged from slightly round-edged particles. Another feature that can be observed in the SEM images is the consistency of the NSs. The IND + F68 NS have the most homogenous composition compared to the other NSs (Fig. 3C-D). IND + P1 (Fig. 3G-H) and IND + P2 NSs (Fig. 3I-J) appear to have a slightly higher variation in particle size compared to F68.

### Dissolution Studies

**Fig. 4:**
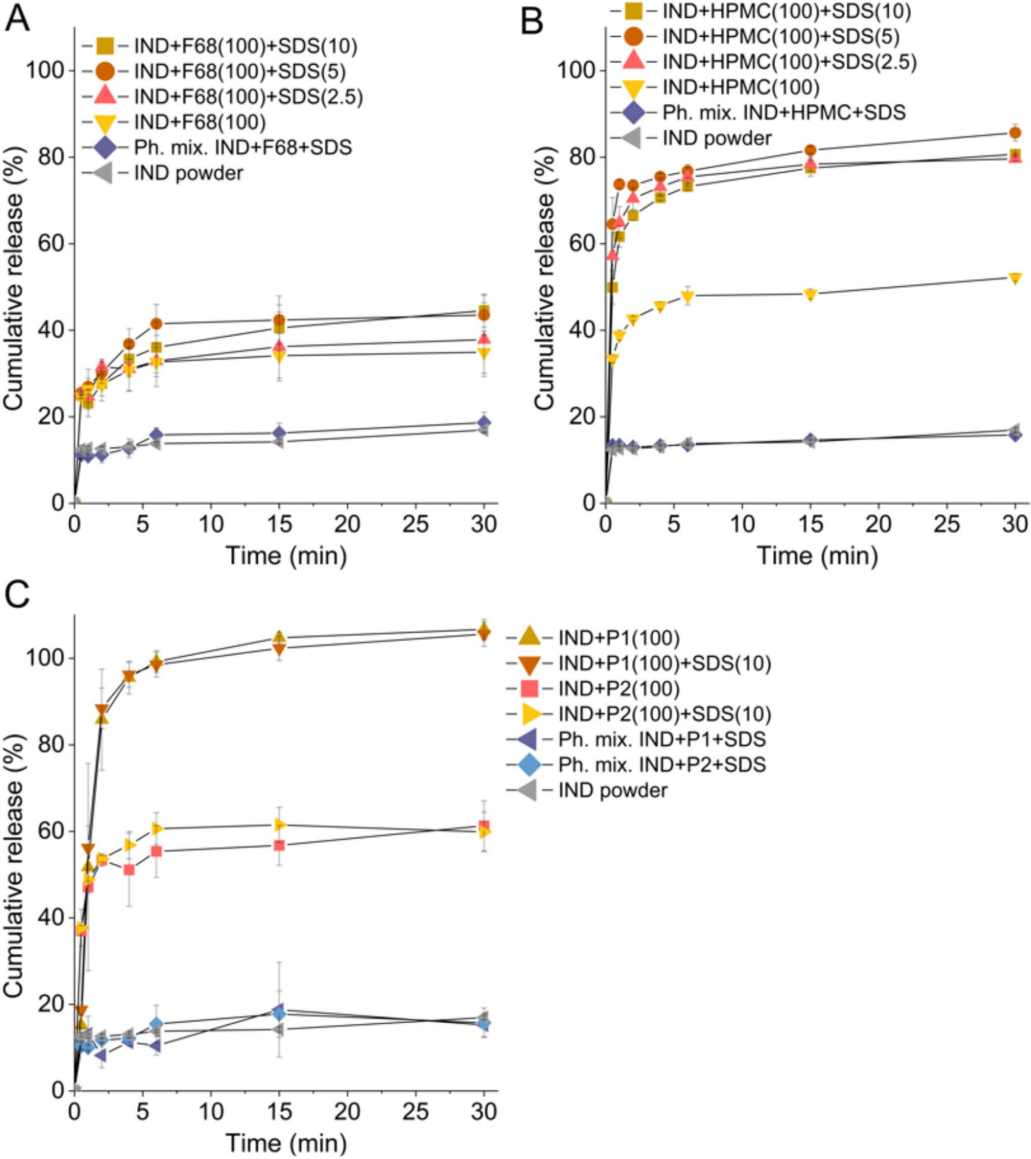
Dissolution profiles of NS formulations and reference samples (*n* = 3) performed under non-sink conditions. (**A**) F68 (**B**) HPMC, and (**C**) P1 and P2 polymer containing samples.

The dissolution tests were performed on day 1 after preparation of the NSs to exclude any possible variations caused by differences in particle size as all the samples were mostly in the same particle size range at this time point. Dissolution studies under non-sink conditions showed that the reference samples, including IND powder and physical mixtures, have the same solubility properties and profiles, *i.e.*, the specific drug release level was achieved within the first 5 min and the cumulative drug release was less than 20% (Fig. 4). This finding confirms that the mere addition of stabilizing agents to the powder sample composition did not improve the solubility of IND. Phthalate buffer at pH 5 was employed as the dissolution medium not to mimic physiological relevance, but rather to create unfavorable conditions for IND dissolution and thereby highlight potential differences between the formulations.

The dissolution profile of the NSs for each stabilizer reached a certain drug release level depending on the composition of the formulation. The addition of SDS significantly affected the dissolution properties only for the HPMC-stabilized NSs (*p* < *0.05*) (Fig. 4B). HPMC is well-known for its use as a matrix excipient for controlled release properties [62, 63]. It should be noted that, in general, higher-viscosity grades of HPMC are more commonly applied as excipients in controlled-release formulations. In this study, HPMC E5 grade was used, and considering the outcome, the reason behind the dissolution results for HPMC formulations is caused by a HPMC matrix on top of the drug particle. In other words, HPMC formulation without SDS seems to cover the drug particle surface with a controlled release coating leading to a more restrained dissolution of the drug. On the other hand, the addition of SDS into the HPMC formulation eliminates or disperses the controlled release shell and enables the drug nanoparticle to solubilize more efficiently. With 30–40% cumulative drug release, the NS formulations with F68 presented the lowest value of all polymer stabilized formulations, even with the addition of SDS (Fig. 4A). HPMC showed a ca. 50% cumulative release without SDS while in combination with SDS the cumulative release increased to 80%. The dissolution profiles of P1 and P2-stabilized NSs significantly differed from each other (*p* < *0.05*) (Fig. 4C). The P2 polymer showed a cumulative drug release of ca. 60%, whereas the P1 polymer reached a 100% cumulative drug release. Furthermore, the addition of SDS to P1 and P2-stabilized NSs did not affect the cumulative drug release. To possibly gain a better understanding for the different dissolution profiles of P1 and P2-stabilized NSs, we conducted molecular dynamics simulations.

### Molecular Dynamics Simulations

The MD simulations were designed to investigate the interactions between the IND drug crystal and two ABA-type triblock copolymers with all atom resolution: P1 (with a BuOzi middle block) and P2 (with a BuOx middle block). The qualitative nature and relative strength of polymer–crystal interactions were assessed through trajectory visualization and calculation of R_g_, RMSD, SASA, contact analysis, and mass density profile measurements. This qualitative insight provided by the MD simulations can be used to elucidate the mechanism behind the quantitative results of the experiments. While the results are approximate, and the model used has limitations, the behavior of the two polymers differs sufficiently, in a qualitative fashion, that the conclusions made from the simulations can be justified.

It was found that the R_g_, RMSD and SASA of the polymer were higher for P1 than for P2, primarily reflecting differences in molecular weight and polymer architecture (Table I). Importantly, the standard deviation of R_g_, RMSD and SASA were larger for P2, indicating greater conformational flexibility and fluctuation. These data suggest that P2 undergoes more dynamic rearrangements at the interface, while P1 adopts a comparatively more persistent adsorbed state.

**Table I:** Comparison of average R_g_, RMSD, and SASA for the P1 and P2 polymers interacting with the IND drug crystal for the last 100 ns of simulations

Polymer	R_g_ (nm)	RMSD (nm)	SASA (nm^2^)
P1 (with a BuOzi middle block)	1.34 ± 0.01	1.152 ± 0.008	45.0 ± 0.6
P2 (with a BuOx middle block)	1.22 ± 0.03	1.08 ± 0.02	43 ± 1

**Fig. 5:**
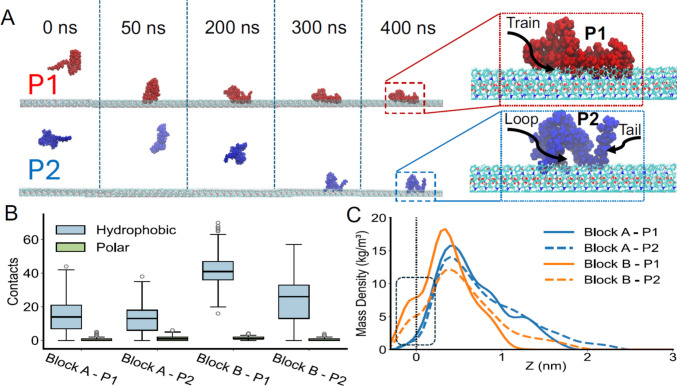
(**A**) Molecular dynamics simulation snapshots. The images show the crystal surface and the polymers at different times from 0 to 400 ns. Polymer P1 is shown in red and polymer P2 is shown in blue (water molecules are not shown). The right-side images show a magnified image of the IND drug crystal and polymers at the end of the simulations at 400 ns. Polymer P1 is shown on the top image and polymer P2 is shown on the bottom image. (**B**) Blockwise contact analysis of polymer P1 and P2 for hydrophobic contacts and polar contacts. (**C**) Blockwise mass density profile of P1 and P2. The black dotted frame near the origin highlights the penetration of polymers below the zero reference, *i.e.*, below the surface chlorine atoms, showing the pronounced penetration of the P1 polymer into the grooves of the crystal surface.

Trajectory snapshots at 0 ns, 50 ns, 200 ns, 300 ns, and 400 ns revealed distinct adsorption behaviors on the IND drug crystal for the two polymers (Fig. 5A). Initially dispersed in the aqueous phase, both P1 and P2 migrated toward the IND drug crystal surface. P1 quickly adsorbed, gradually flattening onto the crystal surface. Both the hydrophilic (Block A) and hydrophobic (Block B) segments of P1 contributed to crystal surface association, with the polymer adopting a compact conformation in close proximity of the crystal surface. In contrast, P2 displayed more unstable or dynamic surface interactions with slower adsorption to the IND drug crystal surface. Even after adsorption, it retained a looped conformation and exhibited persistent structural fluctuations. VMD snapshots at 400 ns highlight these differences, with P1 forming a well-adhered, closely surface associated configuration and P2 remaining loosely associated and partially elevated above the surface.

Furthermore, contact analysis was performed to quantify hydrophobic and polar interactions between the polymers and the IND drug crystal within a 0.4 nm cutoff distance. In both systems, hydrophobic interactions dominated (Fig. 5B). For P1, Block B contributed with the largest number of hydrophobic contacts, which is consistent with its more hydrophobic and bulkier BuOzi core. P2 showed fewer total contacts, for both Block A and B. Polar contacts remained low and comparable for both polymers. These trends point to more frequent and persistent interfacial contacts for P1, which is driven by enhanced hydrophobic interactions.

Mass density profiles along the z-axis (Fig. 5C) showed that, for both polymers, Block B is located near the crystal surface and Block A extends into the aqueous phase. However, P1 displayed higher and sharper density peaks for both blocks near the surface, which indicates closer surface association. In contrast, P2 showed broader profiles with density distributed more into the solvent, which is consistent with weaker surface interactions. The mass density profile highlights that P1 penetrates deeper into the crystal surface, specifically into the grooves below the zero reference level, compared to P2. This indicates that P1 displays closer surface adsorption than polymer P2. Altogether, P1 adopts a more surface-associated configuration lying relatively flat on the IND drug crystal surface and penetrating the surface, while P2 displays transient contacts separated by loops extending away from the surface.

### Stability Studies

The physical stability of NS formulations complying with the target particle size and PdI was measured for 28 days. In this case, colloidal stability was evaluated as the ability to avoid aggregation and sedimentation, *i.e.*, to maintain their particle size and PdI, and evaluating any changes in the ζ-potential.

**Fig. 6:**
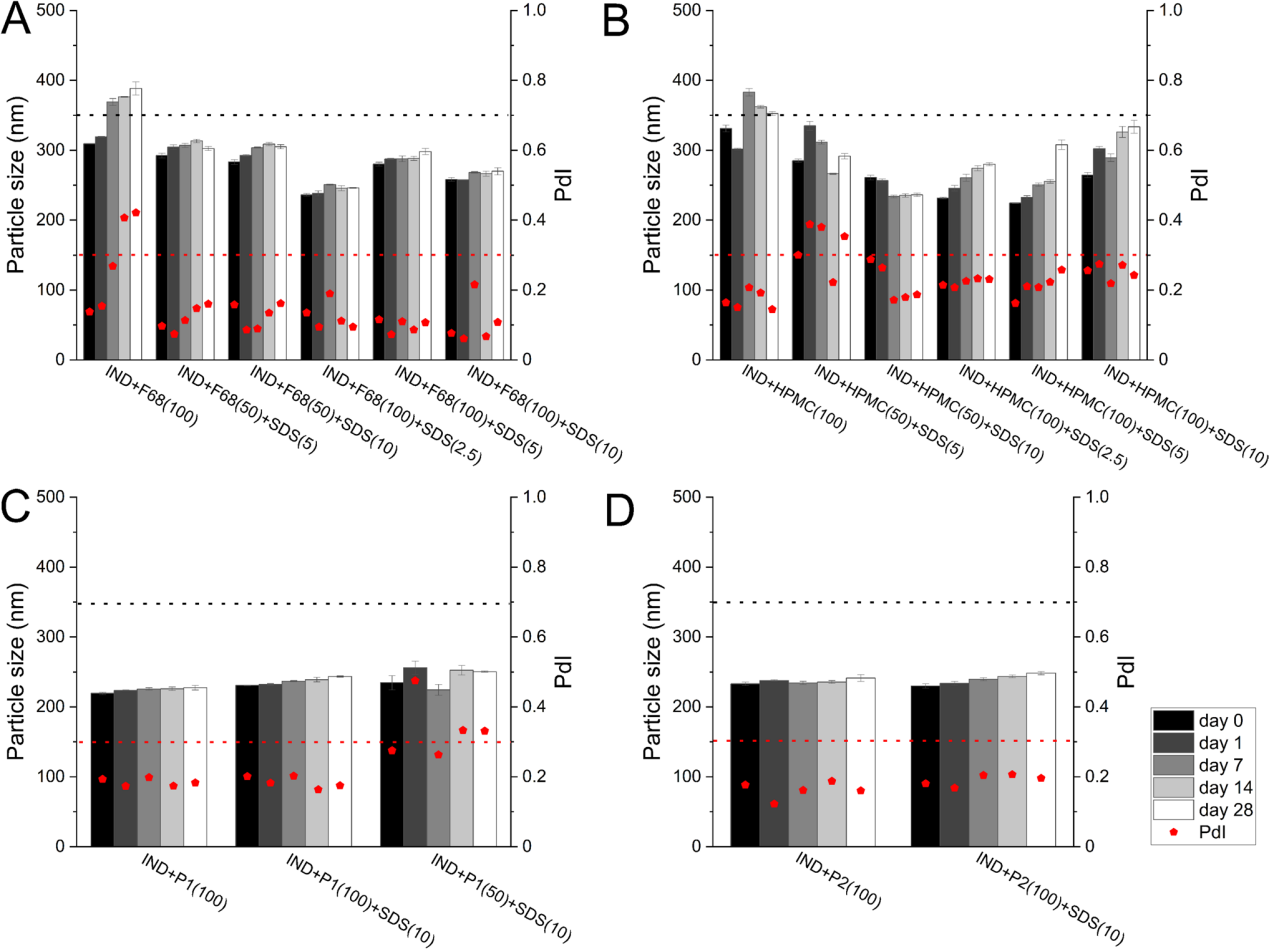
Stability of F68 (**A**), HPMC (**B**), P1 (**C**) and P2 (**D**) NS formulations up to 28 days of storage (*n* = 3). Dashed horizontal lines represent the target values for particle size (black) and PdI (red).

The PdI values for IND + F68(100) and IND + HPMC(100) NSs indicates that their stability slightly decreased already after 7 days (Fig. 6). Previously it has been shown that the addition of SDS in the formulation improves the size reduction and stability in the case of F68 and HPMC NS formulations (Figure S1). Accordingly, our data shows that SDS clearly improved the stability of the F68 and HPMC NS formulations up to 28 days, albeit more effectively for F68 (Fig. 6). The IND + F68(50) and IND + HPMC(50) formulations with added SDS complied with the target values and showed good stability for up to 28 days, indicating that the synergistic stabilizing effect of SDS is practically independent of the amount of F68 and HPMC stabilizer, if the amount used equals 50 mg or greater.

The NS formulations IND + P1(100) and IND + P2(100) presented with a slightly different behavior. Here, the addition of SDS did not notably affect the particle size, and the stability of the NSs did not vary between the formulations with or without SDS. A consistent quality and stability with suitable particle sizes of the NSs were achieved in both cases, which was also supported by reasonable low and stable PdI values. In contrast, lowering the amount of P1 down to 50 mg and adding SDS (10 mg) presented different results compared to P2. In case of IND + P1(50) + SDS(10), the target characteristics set in advance were met, thus this formulation was also studied for the stability properties, and confirmed good stability for 28 days. As IND + P2(50) + SDS(10) did not meet the target values, its stability was not further evaluated.

Interestingly, it can be noted that the ζ-potential of IND + P1(100) and IND + P2(100) was positive in contrast to the negative values observed with the other stabilizers (Figure S3). SDS is anionic, but no significant differences could be seen in the ζ-potential values with added SDS, as the amount of SDS is rather low in all cases [64]. In contrast, for IND + P1(50) + SDS(10) the ζ-potential decreased down to close to a zero level. It is often assumed that when the ζ-potential value is highly positive or negative, electrostatic repulsion between particles is sufficient to prevent sedimentation or aggregation of the particles – provided that stabilization is primarily charge-based [65]. However, in this study, the stability of the NS formulations is primarily attributed to steric stabilization by polymeric stabilizers adsorbed on the surface of the drug nanocrystals. Therefore, electrostatic repulsion plays a minimal role in maintaining colloidal stability.

## Discussion

Our study confirmed that the POzi and POx-based polymers are effective as stabilizing excipients in formulating drug NS formulations. P1 and P2 were able to achieve stable nanoparticle sizes for IND NSs. The novel NS stabilizing excipients P1 and P2 sustained control over particle size during the 28-day stability testing period (Fig. 6). In addition, stability studies did not reveal any signs that the stability of these NSs could be compromised for longer storage periods.

Besides maintaining excellent physical stability, the resulting particle size with P1 and P2 stabilization was significantly smaller compared to F68 and HPMC-stabilized NSs. The reduction in particle size is a significant factor of potential improvement of solubility properties and maximum solubility of the API. In this case, this was also confirmed by the dissolution evaluation carried out in this study. Dissolution tests highlighted clear differences in the cumulative drug release profiles between the NS formulations and the reference samples (Fig. 4). All reference samples settled at the same cumulative drug release level of ca. 20%, while the P1 and P2 polymer stabilized NSs achieved cumulative drug release levels of 100% and 50%, respectively. Furthermore, F68 and HPMC stabilized NSs reached cumulative drug release levels between 60–80%. This confirms that the difference between the reference samples and the corresponding polymer stabilized NSs is primarily due to the reduction in particle size. The components used in the NS formulation did not significantly affect the dissolution profiles of IND when prepared as a physical mixture. The overall dissolution results show that all polymer-stabilized NS formulations dissolve more rapidly, reaching higher cumulative release levels compared to the reference samples. The exceptional advantage of the P1-stabilized NSs was that they achieved a cumulative drug release level of 100% after 15 min in the dissolution tests. On the other hand, P2-stabilized NSs reached a 50% cumulative drug release level of IND, which is also a significant improvement when compared to the pristine IND powder as the dissolution test is performed in non-sink conditions. It should be noted that the only difference between P1 and P2 is one methylene group per repeat unit in the backbone of the polymer.

The addition of SDS did not change the outcome of the stability for the P1 and P2 formulations as it did with the traditional stabilizers, which can be considered a significant advantage to avoid excessive use of different excipients in the formulations. Moreover, HPMC and F68 NSs were more dependent on the addition of SDS to achieve particle size reduction in the first place. The dissolution profiles showed that most stabilizer combinations reached a certain stabilizer dependent level of drug release, regardless of SDS addition, except for HPMC. However, in the case of HPMC, even a small addition of SDS into the formulation resulted in a clear increase in the cumulative drug release level compared to the pure HPMC NS formulation. This difference is likely due to the inherent gelling tendency of HPMC, a property frequently utilized in controlled-release formulations [62]. In fact, HPMC + SDS formulations resulted in a better cumulative release of IND compared to the P2 formulations. When evaluating the NSs prepared in this study, it must be considered that IND is a class II drug according to the BCS, *i.e.*, it is a poorly water-soluble drug with high permeability [66]. Therefore, even a modest increase of about one-tenth in dissolution level under non-sink conditions could positively influence the *in vivo* bioavailability of IND.

The combined DSC and LF-Raman results confirmed that no unexpected structural changes occurred in the crystal structure of the API during the milling process (Fig. 2), *i.e.*, no formation of a different polymorph nor amorphous form was detected after milling. DSC measurements showed a small shift in the IND melting peak, but the ɑ polymorphic form of IND would show a melting point onset at less than 140 °C, thus a formation of the ɑ-form is ruled out [61]. A more likely explanation for the small changes in the melting peak behavior is the overall impact of the formulation, the smaller particle size and the probability of the drug to dissolve into the stabilizer which is a commonly known phenomenon called melting point depression [55]. Nevertheless, due to the onset shift in the DSC thermogram, a definite statement based only by evaluation of the DSC results was deemed not reliable. Therefore, the LF-Raman study was added to confirm the polymorphic form of IND in our formulations, as LF-Raman is a particularly accurate qualitative tool for such studies [61]. In the Raman spectra, the ɑ-form presents a peak at 1649 cm^−1^, which was not observed in any of the formulations studied [67]. LF-Raman spectra together with the DSC results thus confirmed that the powder reference samples as well as the NS formulations represented the same γ-form of IND, which is the most common polymorphic form of IND [59].

The findings in the ζ-potential measurements corroborated the other results. In the NS formulations with traditional stabilizers, the ζ-potential would predict slightly lower stability for HPMC stabilized NSs as the surface charge nearly halved compared to F68 stabilized NSs. Therefore, NSs stabilized with HPMC had a more pronounced tendency to increase in particle size during storage. The stabilizing effect of HPMC is known to be based on its property of viscosity enhancement whereas F68 and other poloxamers tend to adsorb on the surface of the drug crystal and thus forming an intact steric stabilizing layer [68–70]. SEM images of the NS formulations (Fig. 3) support that the P1 and P2 polymers behave in the same manner as F68 in this formulation strategy. Consequently, it can be estimated that they would result in a prolonged circulation time in the blood stream after intravenous administration, as well as metabolic stability when administered orally [71]. Unlike F68 and HPMC, the NSs stabilized by P1 and P2 had a strong positive surface charge. In general, a positive surface charge has been associated with an increased toxicity and increased phagocytosis on several types of nanosystems [72, 73]. At the same time, there has been some reports indicating that positive surface charge can have some advantages in drug delivery systems to achieve a desired biological response [74–76]. Previous studies on the safety and biocompatibility of POzi and POx polymers have shown excellent benchmark regarding these factors, and structurally slightly different POzi and POx-based polymer-drug conjugates have reached clinical trials [25, 26, 77, 78]. This suggests that the POzi and POx polymers used in this study would be very attractive polymers to be used as stabilizers for NSs.

As mentioned before, Ramsey *et al.* [30] have already demonstrated the use of POx-based polymers as stabilizing agent in drug suspensions. More precisely, their study included P2 to prepare drug suspensions for aerosol delivery. The study reports the particle sizes in the sub-micron range, however, probably due to the low amount of stabilizer used, the particle size quickly increased up to micron size range right after the preparation day which indicates a high tendency for aggregation. Thus, in contrast to our findings, they cannot be considered as a stable NS. We tentatively attributed the different results to the differences in the ratio between the drug and stabilizer amounts, and differences in the sample preparation methods. In our study the highest amount of the P1 and P2 (100 mg) polymers was 8% by weight of API amount, whereas for the inhalable formulation in the Ramsey *et al.* study a 6.6% by weight of API was used. This difference affects the strength of steric stabilization and is directly related to achieving a stable NS formulation. It is also possible that the observed difference results from the use of a different API. In any case, this study shows for the first time the use of P1 and P2 polymers as stabilizers for highly stable IND NSs with sizes below 300 nm which preserves their size for at least 28 days. Furthermore, this study specifically shows that P1 performed better than P2, albeit both polymers compare favorably to the traditionally used stabilizers F68 and HPMC.

P1 clearly distinguishes itself from traditional stabilizers (F68 and HPMC) by forming much more stable NSs with smaller particle sizes. The NS formulations with P2 also formed NSs with smaller particle sizes and showed clearly improved stability compared to the traditionally used stabilizers. Although they did not reach the same dissolution levels as P1 and HPMC + SDS formulations in the non-sink *in vitro* dissolution tests, it is possible that under *in vivo* sink conditions, they may still dissolve rapidly and completely. In addition, the performance of the NSs stabilized with 100 mg of P1 and P2 were not affected by the addition of 10 mg SDS. This observation indicates that it is feasible to achieve desired characteristics of NSs formulations with the P1 and P2 polymers by using lower amounts and number of used excipients compared to traditional stabilizers, such as F68 and HPMC. At the same time, lower amount of POzi-based polymer (50 mg) complied with the target characteristics when 10 mg of SDS was added into the formulation. This indicates that SDS can play a significant role in formulations with a reduced amount of stabilizing polymer but is insufficient in systems where the stabilizer concentration is already adequate. With sufficient amount of POzi and POx stabilizers, the disadvantages associated with the use of such surfactants in orally administered pharmaceutical formulations could be overcome. Such disadvantages are for example unpleasant taste and potential side effects in the gastrointestinal tract including allergic reactions [62, 79]. These create a real challenge especially in pediatrics, but with the POzi and POx-based polymers used in this study, this challenge might be addressable.

The MD simulations suggest that P1 shows a more persistent surface association with the IND drug crystal in water compared to P2. This enhanced interaction is primarily driven by hydrophobic interactions, particularly by Block B. While both polymers share the same hydrophilic Block A (MeOx), the difference in Block B chemistry appears to play a crucial role. The BuOx block of P2 contains one fewer methylene group (–CH_2_–) per monomer, thus leading to reduced hydrophobicity and flexibility. Both factors might contribute to the observation of fewer contact points with the IND drug crystal surface.

The increased flexibility of the polymer appears to make it easier for neighboring hydrophobic side chains to fit into the grooves on the crystal surface. Contact analysis and mass density profile indicate that hydrophobic interactions dominate, whereas polar interactions remain minimal for both polymers. The polar regions of the polymer instead orient away from the surface, thus reducing the effective hydrophobicity of the crystal surface.

Previously, Liu *et al.* studied experimentally the interactions of block co-polymers of Pluronics with IND drug nanocrystals using surface plasmon resonance and contact angle measurements; they interpreted their results as being examples of what they described as ‘train’-like stable conformations or ‘loops’ and ‘tails’ above the surface [80]. In line with these experiments, the MD simulations in this study also suggest that the more hydrophobic polymer P1 establishes a more ‘train’-like conformation during adsorption onto the crystal surface via the BuOzi block, while the P2 polymer forms more ‘loops’ and ‘tails’ above the surface. The loops and tails try to extend into the aqueous phase as the flattening of the polymer on the crystal surface is entropically unfavored. Additional simulations of Pluronic F68, referred to as P3 (Figures S5–S6 in the Supplementary material) show adsorption of P3 on the crystal surface with a more extended conformation and reduced hydrophobic contacts compared to P1, which is consistent with weaker surface association of P3.

Altogether, the MD simulation results suggest a mechanism through which P1 acts as a better dissolution agent than P2. The backbone of P1 is both more hydrophobic and more flexible than that of P2, enabling better contact between hydrophobic side chains and the grooves of the crystal surface. This results in continuous coverage of the hydrophobic surface, while polar groups of the polymer remain exposed. In essence, this produces a efficient detergency-like effect, enhancing drug dissolution to a higher plateau level. These observations from MD simulations offer a qualitative insight into the molecular-level interpretation of the experimentally observed dissolution profiles.

## Conclusions

In this study, the formulation and characterization of NSs utilizing POzi and POx-based triblock copolymers as stabilizers were studied and compared with traditionally used stabilizers, namely HPMC and F68. Several key points regarding the potential use and characteristics of these experimental excipients in NS formulations were observed. This study demonstrated that by using these polymers and by a careful formulation optimization approach, it was possible to avoid using an additional excipient, SDS, for preparing viable NSs. The novel stabilizers P1 and P2 demonstrated excellent stabilization properties compared to traditional stabilizers. Above all, the novel NS stabilizers showed minimal particle size variation even without SDS, highlighting their natural stabilizing efficacy. Among the two slightly different novel polymer stabilizers, P1 turned out to be particularly promising especially by presenting exceptional performance in dissolution tests when compared to the other stabilizers used. MD simulations allowed us to evaluate how the molecular interactions influence the properties of our formulations, especially the dissolution of the drug. This emphasizes its potential for enhancing drug release properties and solubility enhancement in orally administered NS formulations. Moreover, this study revealed that SDS did not provide any significant benefits when incorporated with P1 and P2, thus challenging conventional beliefs regarding its advantages when used in NS formulations. Nevertheless, POzi and POx-based polymers are not listed in any pharmacopoeia, and their use as excipients would be subject to regulatory assessment as part of a pharmaceutical product application. Further studies could also consider the suitability of these polymers as stabilizers for NSs of other types of APIs to ensure their wide applicability and versatility in NS-based drug delivery systems.

## Supplementary Information

Below is the link to the electronic supplementary material.

ESM1 (DOCX 1.67 MB)

## Data Availability

All data of the results of this study are available within the article and its Supplementary Information. Any additional information is available from the corresponding author upon reasonable request.

## Abbreviations

API: Active pharmaceutical ingredient
BCS: Biopharmaceutical Classification System
BuOx: 2-N-butyl-2-oxazoline
BuOzi: 2-N-butyl-2-oxazine
DLS: Dynamic light scattering
DSC: Differential scanning calorimetry
HPMC: Hydroxypropyl methylcellulose, hypromellose
IND: Indomethacin
LF: Low frequency
MD: Molecular dynamics
MeOx: 2-Methyl-2-oxazoline
NS: Nanosuspension
PdI: Polydispersity index
Pox: Poly(2-oxazoline)
POzi: Poly(2-oxazine)
R_g_: Radius of gyration
RMSD: Root-mean-square deviation
SASA: Solvent-accessible surface area
SDS: Sodium dodecyl sulfate
F68: Pluronic F68, Poloxamer 188
