# Supplementary material for: Formulation and Evaluation of Indomethacin Nanosuspensions Stabilized by Poly(2-oxazine) and Poly(2-oxazoline)-Based Polymers for Solubility Enhancement
Source: Pharm Res. 2026 May 15;43(5):1691–709. doi: 10.1007/s11095-026-04109-0 (PMC13269528; doi:10.1007/s11095-026-04109-0)
Supplement: Supplementary file 1 — (DOCX 1.67 MB) [file 11095_2026_4109_MOESM1_ESM.docx]

**Supplementary material**

**Formulation and Evaluation of Indomethacin Nanosuspensions Stabilized by Poly(2-oxazine) and Poly(2-oxazoline)-Based Polymers for Solubility Enhancement**

**Erika Espo^a,^***, Dipti Potdar^b^, Kiana Baas^a,c^, Larissa Keßler^d^, Mengshi Yang^e^, Kārlis Bērziņš^f^, Marianna Kemell^g^, Topias Kiiskinen^a^, Anni Heinonen^a^, Josef Kehrein^a,d,h^, Ben J. Boyd^f,i^, Anssi-Pekka Karttunen^a^, Leena Peltonen^a^, Robert Luxenhofer^d^, Alex Bunker^b^, Tapani Viitala^a,j,^*

^a^Division of Pharmaceutical Chemistry and Technology, Faculty of Pharmacy, University of Helsinki, FI-00014, Helsinki, Finland

^b^Division of Pharmaceutical Biosciences, Faculty of Pharmacy, University of Helsinki, FI-00014, Helsinki, Finland

^c^Laboratory of Pharmaceutical Technology, Department of Pharmaceutics, Ghent University, Ottergemsesteenweg 460, 9000 Gent, Belgium

^d^Soft Matter Chemistry, Department of Chemistry, and Helsinki Institute of Sustainability Science, Faculty of Science, University of Helsinki, FI-00014, Helsinki, Finland

^e^Lehrstuhl für Chemische Technologie der Materialsynthese, Department of Chemistry and Pharmacy, Julius-Maximilians-University Würzburg, Röntgenring 11, 97070 Würzburg, Germany

^f^Department of Pharmacy, Faculty of Health and Medical Sciences, University of Copenhagen, Copenhagen 2100, Denmark

^g^Department of Chemistry, Faculty of Science, University of Helsinki, FI-00014, Helsinki, Finland

^h^Institute of Pharmacy and Food Chemistry, University of Würzburg, Am Hubland, 97074 Würzburg, Germany

^i^Drug Delivery Disposition and Dynamics, Monash Institute of Pharmaceutical Sciences, Monash University, 3052 Victoria, Australia

^j^Pharmaceutical Sciences Laboratory, Faculty of Science and Engineering, Åbo Akademi University, FI-20500, Turku, Finland

***Corresponding authors**: Division of Pharmaceutical Chemistry and Technology, Faculty of Pharmacy, University of Helsinki, FI-00014, Helsinki, Finland. E-mail address: [erika.espo@helsinki.fi](file:///C:\\Users\\apkarttu\\AppData\\Local\\Microsoft\\Windows\\INetCache\\Content.Outlook\\TEA22X4W\\erika.espo@helsinki.fi) (E. Espo) and [tapani.viitala@helsinki.fi](file:///C:\\Users\\ljpelton\\AppData\\Local\\Microsoft\\Windows\\INetCache\\Content.Outlook\\4XU4PIFC\\tapani.viitala@helsinki.fi) (T. Viitala).

Table S1. Stabilizer compositions of NS formulations used in this study.

| NS formulation | Stabilizer (mg) | Surfactant (mg) |
| --- | --- | --- |
| IND+F68(100) | 100 | none |
| IND+F68(100)+SDS(2.5) | 100 | 2.5 |
| IND+F68(100)+SDS(5) | 100 | 5 |
| IND+F68(100)+SDS(10) | 100 | 10 |
| IND+F68(50) | 50 | none |
| IND+F68(50)+SDS(2.5) | 50 | 2.5 |
| IND+F68(50)+SDS(5) | 50 | 5 |
| IND+F68(50)+SDS(10) | 50 | 10 |
| IND+F68(25)+SDS(2.5) | 25 | 2.5 |
| IND+F68(25)+SDS(5) | 25 | 5 |
| IND+F68(25)+SDS(10) | 25 | 10 |
| IND+HPMC(100) | 100 | none |
| IND+HPMC(100)+SDS(2.5) | 100 | 2.5 |
| IND+HPMC(100)+SDS(5) | 100 | 5 |
| IND+HPMC(100)+SDS(10) | 100 | 10 |
| IND+HPMC(50) | 50 | none |
| IND+HPMC(50)+SDS(2.5) | 50 | 2.5 |
| IND+HPMC(50)+SDS(5) | 50 | 5 |
| IND+HPMC(50)+SDS(10) | 50 | 10 |
| IND+HPMC(25)+SDS(2.5) | 25 | 2.5 |
| IND+HPMC(25)+SDS(5) | 25 | 5 |
| IND+HPMC(25)+SDS(10) | 25 | 10 |
| IND+P1(100) | 100 | none |
| IND+P1(100)+SDS(10) | 100 | 10 |
| IND+P1(50) | 50 | none |
| IND+P1(50)+SDS(10) | 50 | 10 |
| IND+P1(25) | 25 | none |
| IND+P1(25)+SDS(10) | 25 | 10 |
| IND+P2(100) | 100 | none |
| IND+P2(100)+SDS(10) | 100 | 10 |
| IND+P2(50) | 50 | none |
| IND+P2(50)+SDS(10) | 50 | 10 |
| IND+P2(25) | 25 | none |


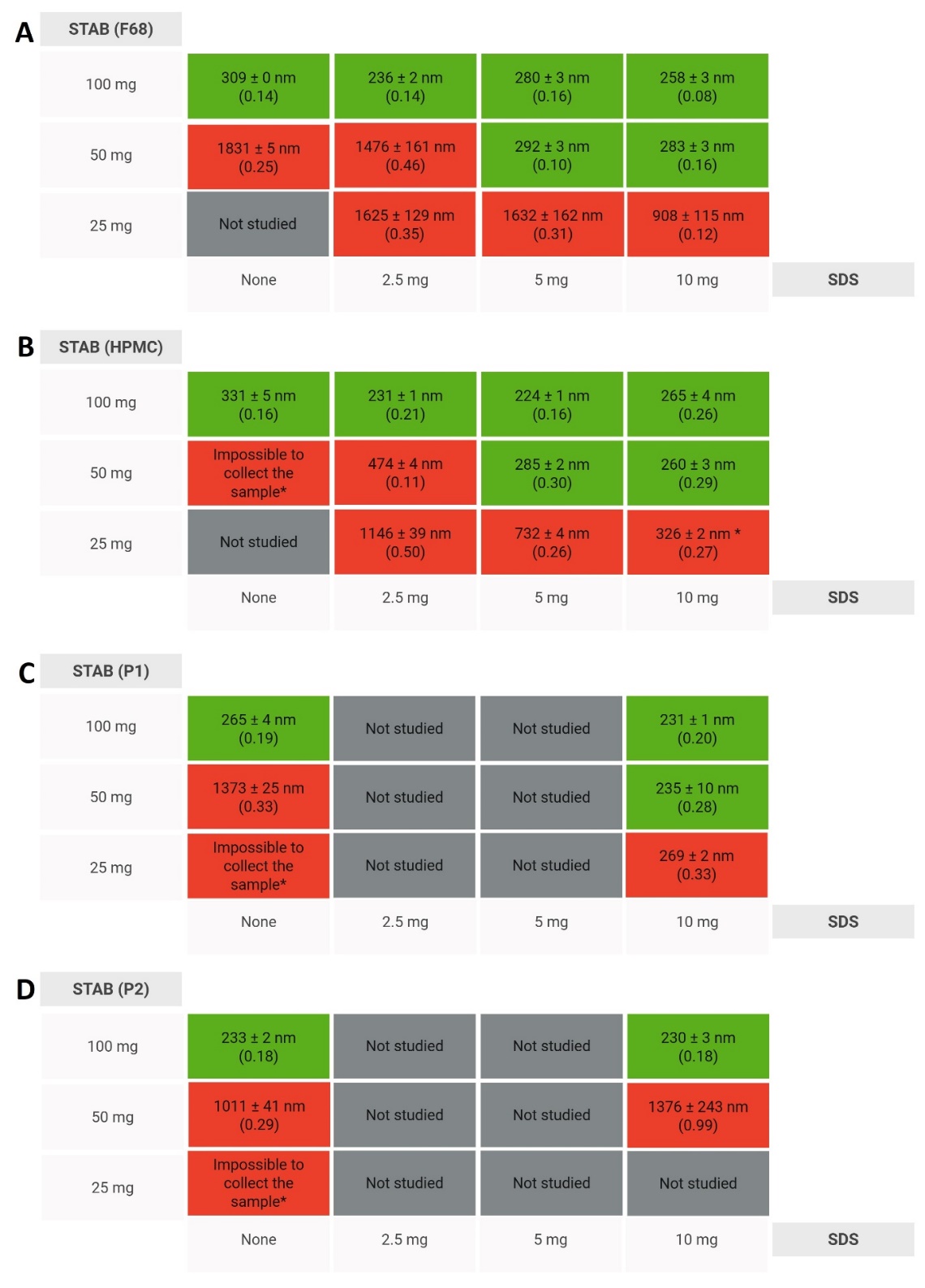


Figure S1. Particle size distributions for NS formulations right after preparation (n=3). The values in parenthesis represent PdI values. A) F68 NS formulations. B) HPMC NS formulations. C) P1 NS formulations. D) P2 NS formulations. *Paste-like consistency, which is considered as non-compliant sample.





Figure S2. DSC thermograms for reference samples (A) and NS formulations on the day of preparation day (B) and day 28 after preparation (C) (n=1). NS samples dried and analyzed as a dry sample. Ph.mix. = physical mixture


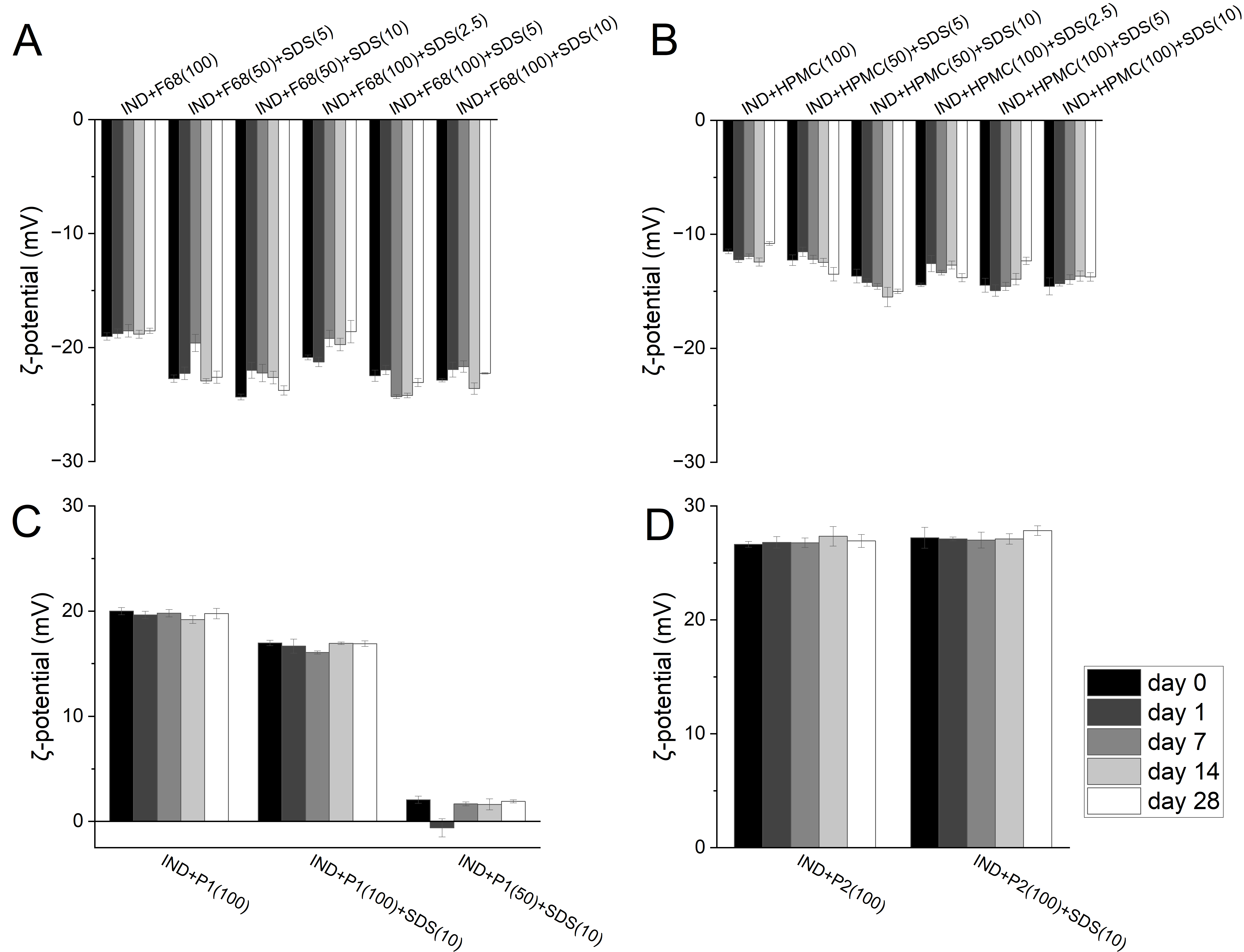


Figure S3. ζ-potential of F68 (A), HPMC (B), P1 (C) and P2 (D) NS formulations up to 28 days of storage (n=3).


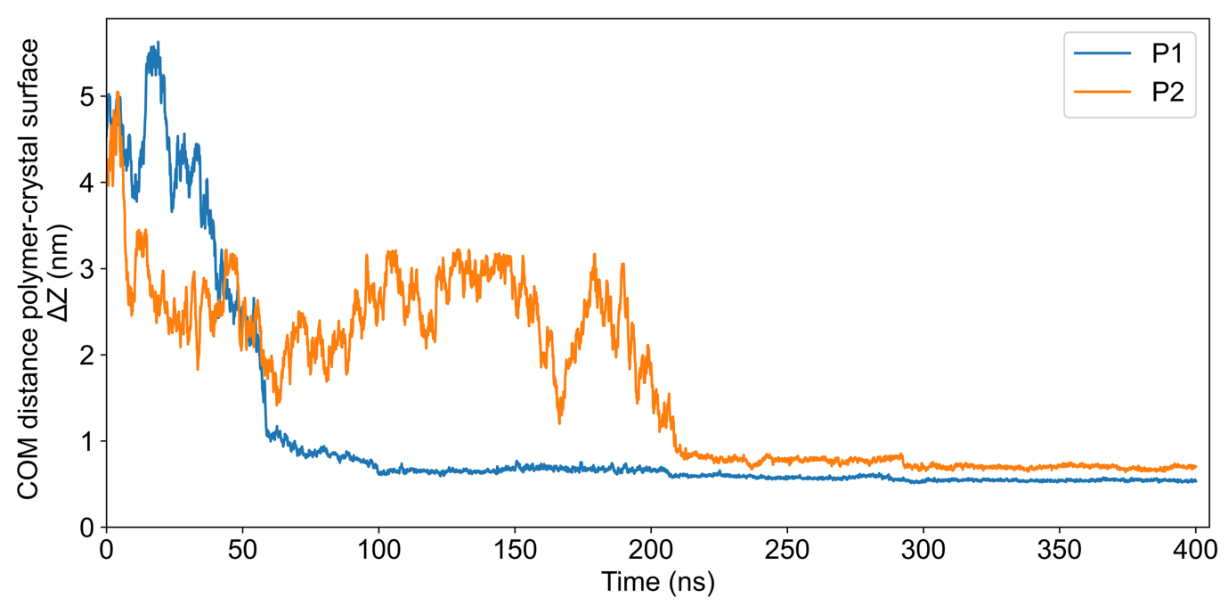


Figure S4. Center-of-mass (COM) distance (ΔZ) between the polymer and the crystal surface for P1 and P2 polymers over 400 ns.

In addition to P1 and P2, simulations were performed for the reference stabilizer P188 or Pluronic F68, denoted as P3 herein (Figures S5-S6). The simulation and analysis methods were the same as was used for the simulation set up of P1 and P2. Here, P3 was modeled as a triblock copolymer (PEO-PPO-PEO) with a block composition of 25-10-25 repeat units, chosen to maintain a comparable chain length with the designed P1 and P2 polymers.


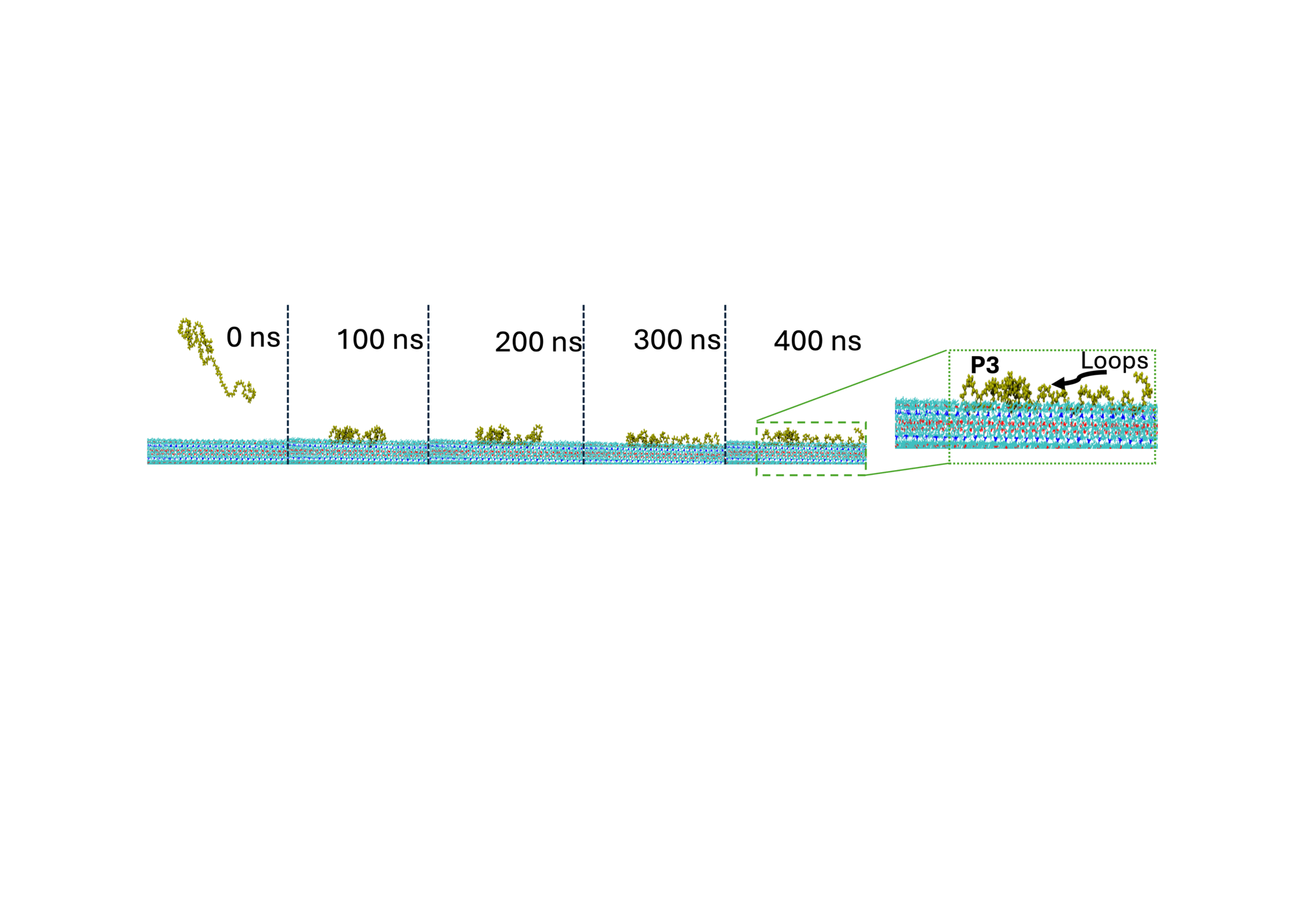


Figure S5. Representative molecular dynamics snapshots of P3 (P188 or Pluronic F68) adsorption onto the crystal surface at different simulation times (0, 100, 200, 300, and 400 ns). The right-most image shows a magnified image of the IND drug crystal and polymer P3 at the end of the simulations at 400 ns.


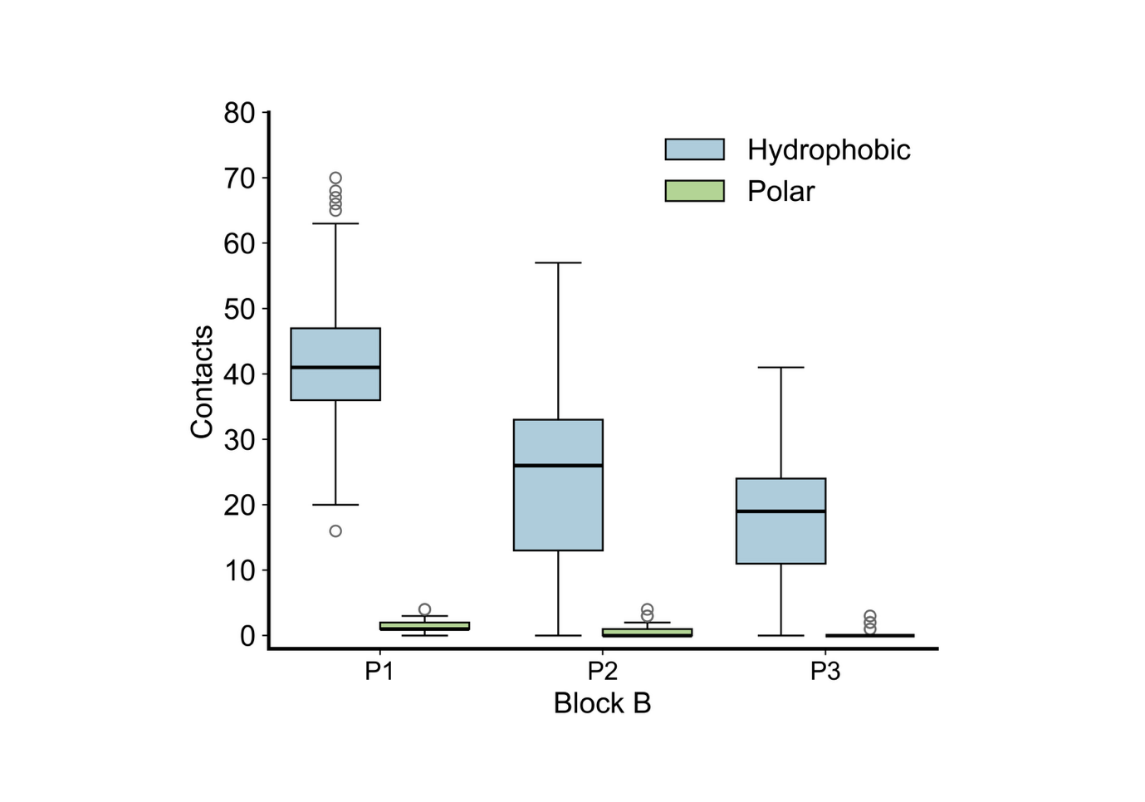


Figure S6. Box plot showing hydrophobic and polar contacts with the crystal surface for Block B of P1, P2, and P3.
